# Supplementary material for: Medication Risks and Their Association with Patient-Reported Outcomes in Inpatients with Cancer
Source: Cancers (Basel). 2024 May 31;16(11):2110. doi: 10.3390/cancers16112110 (PMC11171248; doi:10.3390/cancers16112110)
Supplement: Supplementary file 1 [file cancers-16-02110-s001.zip › cancers-3010951-supplementary.pdf]

Supplementary Information

Medication Risks and Their Association with Patient-Reported Outcomes in Inpatients with Cancer

Maximilian Günther, Markus Schuler, Leopold Hentschel, Hanna Salm, Marie-Therese Schmitz, Ulrich Jaehde

Corresponding author:

Ulrich Jaehde, PhD

Institute of Pharmacy, Department of Clinical Pharmacy, University of Bonn, 53121 Bonn, Germany

u.jaehde@uni-bonn.de

Table S1. Drug classes according to their Anatomical Therapeutical Chemical (ATC) code level 1 and 2 (n = 1884)

ATC-Class	Number	Percentage
**Alimentary system and metabolism (A)**	**551**	**29.2**
Stomatologics (A01)	75	4.0
Remedies for acid-related diseases (A02)	113	6.0
Remedies for functional gastrointestinal disorders (A03)	34	1.8
Antiemetics and anti-nausea agents (A04)	168	8.9
Remedies against obstipation (A06)	49	2.6
Antidiarrheal and intestinal antiphlogistics (A07)	25	1.3
Antidiabetics (A10)	24	1.3
Vitamines (A11)	16	0.8
Minerals (A12)	37	2.0
Others	10	0.5
**Blood and hematopoietic organs (B)**	**168**	**8.9**
Antithrombotic agents (B01)	100	5.3
Antianemics (B03)	24	1.3
Blood substitutes and perfusion solutions (B05)	44	2.3
**Cardiovascular system (C)**	**185**	**9.8**
Diuretics (C03)	49	2.6
Beta-adrenoceptor antagonists (C07)	42	2.2
Calcium channel blockers (C08)	19	1.0
Agents acting on the renin-angiotensin system (C09)	48	2.5
Agents affecting lipid metabolism (C10)	20	1.1
Others	7	0.4

ATC-Class	Number	Percentage
**Dermatics (D)**	**5**	**0.3**
Others	5	0.3
**Urogenital system and sex hormones (G)**	**11**	**0.6**
Urologics (G04)	10	0.5
Others	1	0.0
**Systemic hormone preparations excluding sexual hormones and insulin (H)**	**139**	**7.4**
Corticosteroids for systematic use (H02)	136	7.2
Others	3	0.2
**Anti-infectives for systemic use (J)**	**112**	**5.9**
Antibiotics for systemic use (J01)	66	3.5
Antiviral agents for systemic use (J05)	44	2.3
Others	2	0.1
**Antineoplastic and immunomodulatory agents (L)**	**409**	**21.7**
Antineoplastic agents (L01)	372	19.7
Immunostimulants (L03)	24	1.3
Immunosuppressants (L04)	12	0.6
Others	1	0.0
**Musculoskeletal system (M)**	**40**	**2.1**
Gift medications (M04)	25	1.3
Others	15	0.8
**Nervous system (N)**	**158**	**8.4**
Analgesics (N02)	111	5.9
Antiepileptic drugs (N03)	15	0.8
Psycholeptics (N05)	14	0.7
Psychoanaleptics (N06)	17	0.9
Others	1	0.0
**Antiparasitic agents, insecticides and repellents (P)**	**2**	**0.1**
Others	2	0.1
**Respiratory tract (R)**	**36**	**1.9**
Antihistamincis for systemic use (R06)	28	1.5
Others	8	0.4
**Sensory organs (S)**	**3**	**0.2**
Others	3	0.2
**Varia (V)**	**65**	**3.5**
All other therapeutic agents (V03)	52	2.8
Diagnostics (V04)	13	0.7

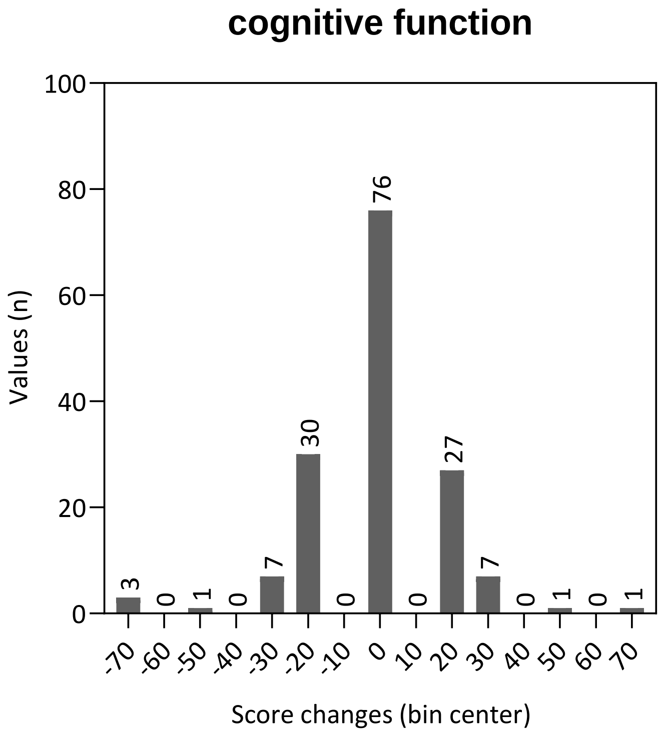

Figure S1. Histogram of the distribution of the score changes in cognitive function from baseline to hospital discharge (n = 153)

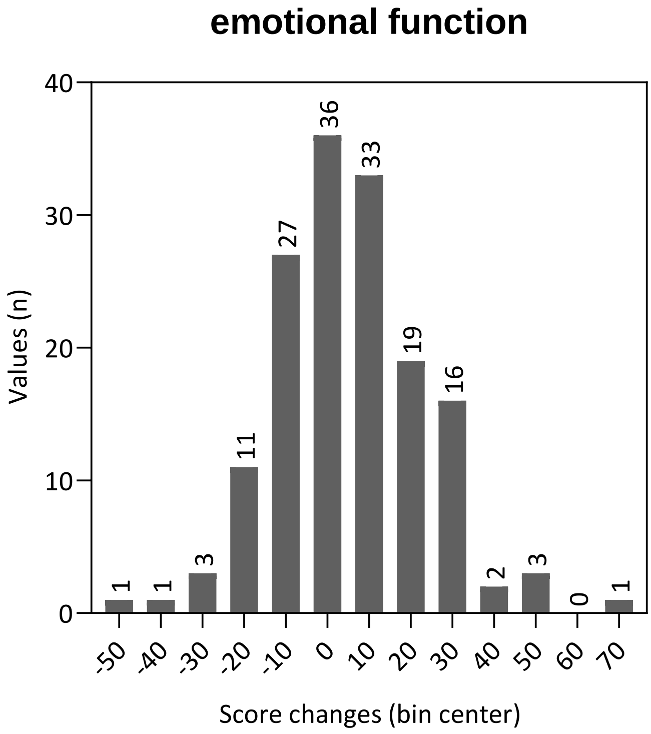

Figure S2. Histogram of the distribution of the score changes in emotional function from baseline to hospital discharge (n = 153)
